# New Spotted Fever Group *Rickettsia* Isolate, Identified by Sequence Analysis of Conserved Genomic Regions

**DOI:** 10.3390/pathogens9010011

**Published:** 2019-12-20

**Authors:** Dar Klein, Adi Beth-Din, Regev Cohen, Shirley Lazar, Itai Glinert, Hiba Zayyad, Yafit Atiya-Nasagi

**Affiliations:** 1 Israel Institute for Biological Research, P.O. Box 19, Ness-Ziona 74100, Israel; adib@iibr.gov.il (A.B.-D.); shirleyl@iibr.gov.il (S.L.); itaig@iibr.gov.il (I.G.); 2 The Ruth and Bruce Rappaport Faculty of Medicine, Technion, Haifa 31096, Israel; regevc@laniado.org.il; 3 Infectious Diseases Unit, Sanz Medical Center, Lanaido Hospital, Netanya 42040, Israel; 4 Infectious Diseases Unit, The Baruch Padeh Medical Center, Poriya Hospital, M.P. The lower Galilee, Tiberias 15208, Israel; habuzayyad@pmc.gov.il

**Keywords:** *Rickettsia*, PCR, multiple sequence alignment, *rOmpA*, *gltA*

## Abstract

The clinical features of spotted fever group (SFG) *Rickettsia* induced disease range from a mild to severe illness. The clinical complexity is even greater due to the fact that the disease can be caused by different species with varying degrees of virulence. Current knowledge asserts that the Israeli SFG (ISF) strain *Rickettsia conorii israelensis* is the only human pathogenic SFG member in Israel. Current diagnostic procedures distinguish between SFG and the typhus group rickettsiosis, assuming all SFG-positive clinical samples positive for ISF. Molecular studies on questing ticks over the past decade have uncovered the existence of other SFG strains besides ISF in Israel and the region. This study describes the first documented analysis of SFG-positive samples from Israeli patients with the goal of distinguishing between ISF and non-ISF SFG strains. We managed to identify a new *Rickettsia* isolate from three independent clinical samples in Israel which was shown to be an as-yet unknown SFG member, showing no absolute identity with any known *Rickettsia* species present in the NCBI database.

## 1. Introduction

*Rickettsia* from the spotted fever group are important emerging vector borne infections of humans worldwide. These are obligate intracellular Gram-negative bacteria transmitted by ticks [1]. The different SFG rickettsioses share several clinical features such as fever, headache, and rash, but may vary in disease severity [1], and may have unique features (e.g. local lymphadenopathy [1]), or different frequencies of occurrence (e.g. skin eschar (tache noire) formation at the tick bite site [2]).

Molecular typing of infectious agents is an important tool for better understanding of ecological niches and identifying circulating species and their virulence [3], as well as for epidemiologic and clinical use. In general, along with the development of the molecular typing and sequence analysis techniques the number of newly described SFG rickettsia species has increased [4,5].

Sequence analysis of PCR-amplified fragments of SFG targeting a number of genes has become a reliable and accepted method for the identification of rickettsia species. These genes encode for citrate synthase (*gltA*), *Rickettsia*-specific outer membrane protein (*rOmpA*), 17kDa lipoprotein precursor antigen gene (*17kDa*), as well as ribosomal *16S rRNA* gene, among others [3,6,7,8,9]. The *rOmpA* encoding gene is present among all SFG members, as immune serum to *rOmpA* reacts with all the SFG members. This gene is considered a good candidate for phylogenetic analysis, as it has been shown to exhibit high degrees of interspecies variation [6]. Citrate synthase is a component of nearly all living cells and is a part of a central metabolic pathway, the citric acid cycle, which plays a key role in energy production and in providing important biosynthetic precursors [7].

Previous studies from Israel found that the Israeli SFG *Rickettsia* strain is *R. conorii israelensis*, with substantial antigenic and genetic diversity existing within this strain [10]. Recently however, other SFG rickettsia species have been molecularly identified in samples from questing ticks in Israel, including: *Rickettsia conorii caspia* [11], *Rickettsia massiliae* [2], *Rickettsia africae*, *Candidatus Rickettsia barbariae* [3,12], and others [3,13].

At the national rickettsiosis reference laboratory in Israel, nested PCR assays are the standard molecular diagnostic method for testing clinical samples. PCR methods for detection of *R. conorii* in those samples include a nested assay for the *17kDa*-protein encoding gene. This PCR-assay allows identification of samples as rickettsia members as well as the classification of these positive samples as either SFG or typhi group (TG) rickettsiosis. The sequence used in this diagnostic assay is highly conserved among rickettsiosis species. Similarly, since members of the *Rickettsia* are closely related phylogenetically and have a high degree of *16S-RNA* nucleotide sequence similarity [14], the *16S-RNA* was also not applicable for the differentiation between rickettsia strains. Here, in this study, three positive patients were further analyzed by sequencing of unique regions from two conserved rickettsial genes. These analyses revealed a new *Rickettsia* isolate showing no absolute identity with any known *Rickettsia* species present in NCBI database, which caused disease in three independent patients.

## 2. Results

**Figure 1:**
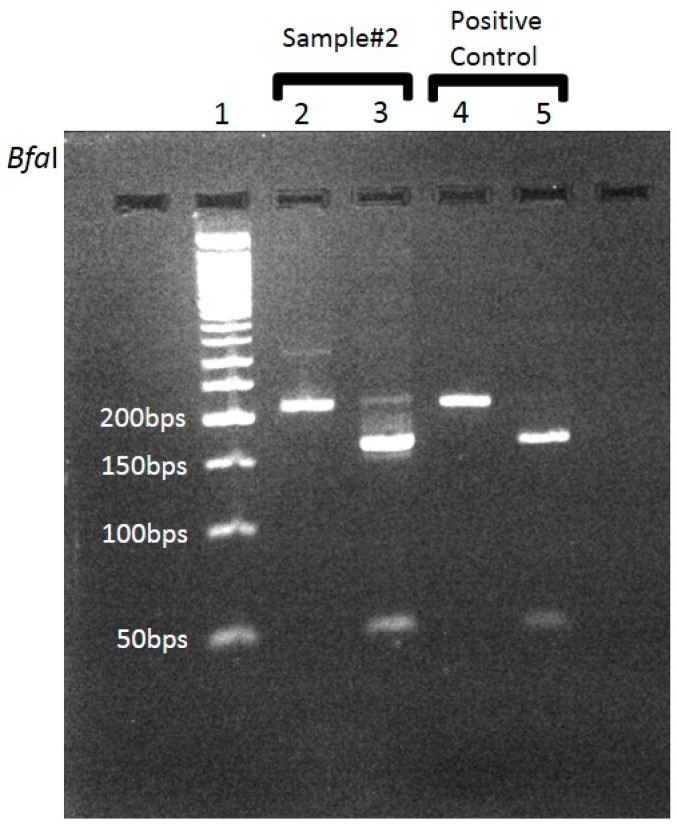
PCR and digestion results of the differential PCR based on the *17kDa*-protein encoding gene. Analysis of PCR products obtained by amplification of the *17KDa* protein’s gene of *Rickettsia conorii israelensis* and patient no.2: 3% agarose gel electrophoresis of restriction endonuclease *BfaI*-digested rickettsial DNA. In lane 2, the nested uncut product positive signal is a 214-bp DNA fragment. Traces of the original band from the external PCR can be seen. *BfaI* digestion results in the generation of two fragments: 164bp and 50bp (lane 3). Traces of uncut product can also be seen. Positive control is *Rickettsia conorii israelensis* DNA. Negative control (no template)―no bands, not shown.

Whole blood samples and skin biopsy specimens from rash lesions taken (during admission) from three different patients suspected to have SFG rickettsiosis were received in our lab for diagnostic PCR assay. These samples were received from three different medical centers, located in distinct geographic regions in Israel, on different occasions. All samples were positive for SFG according to the differential PCR, based on the highly conserved *17kDa*-protein encoding gene (Figure 1) region, using appropriate controls. Following the attribution to the SFG group, these samples were further analyzed separately for determination of the SFG species by an additional PCR. We chose to focus on both *gltA* and *rOmpA* genomic regions. In order to ascertain the validity of this assay, it was previously validated by our group on an array of samples (data not shown). These included human and tick samples, found positive for SFG by the established diagnostic PCR assay. In addition, this assay was tested on DNA purified from known SFG strains (from our strain collection).

BLAST analysis of the *rOmpA* diagnostic region (a 586 basepair region) showed no absolute identity with any known *Rickettsia* species present in NCBI database. Species with high similarity to this isolate having 92.23%–96.89% homology were: *Rickettsia caspia* (GenBank: U83437.1), *Rickettsia slovaca* (GenBank: JX683121.1), *Rickettsia sibirica* (GenBank: U83455.1), and *R. conorii israelensis* (GenBank: U83441.1) (Table 1). Phylogenetic tree analysis was performed using the EMBL–EBI Simple Phylogeny tool. The results did not conclusively allow the assignment of the new strain into an existing sub-group (Figure S1.).

**Figure 2:**
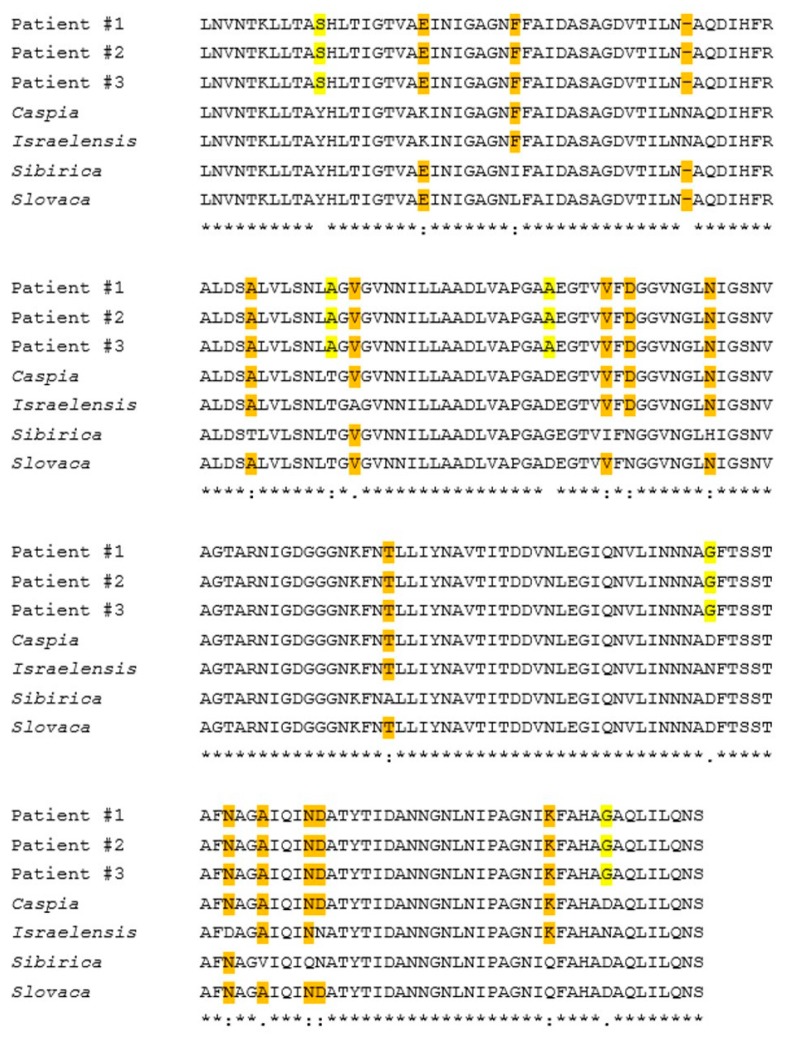
Multiple sequence alignment, for the *rOmpA* protein-containing region of three clinical samples (patients 1–3) and four known *Rickettsia* species. The alignment was performed using EMBL-EBI Clustal W (1.83) with forward primer 120F and reversed primer 760R. *R. caspia* (GenBank accession number AAC35173.1), *R. conorii Israelensis* (GenBank accession number AAC35177.1), *R. sibirica* (GenBank accession number AAC35191.1), and *R. slovaca* (GenBank accession number AFW90551.1). Yellow highlighted = mutations unique to the clinical samples, not appear in any known *Rickettsia* species. Orange highlighted = identical amino acids- showing similarity or difference between the clinical samples and the *Rickettsia* species. Star (*) = similarity. Blank = amino acid from different groups (Non-conservative). Colon (:) = Conservative Amino Acid. Bottom Dot (.) = Semi-conservative Amino Acid.

Multiple sequence alignment of the three samples provides evidence of the perfect match between these samples in the sequenced segments; meaning these samples were identical. When compared to documented *Rickettsia* species we discovered 21 SNP mutations differentiating these samples from the above mentioned species (in the sequenced region) (Figure S2). Of these 21 SNPs, 19 led to changes at the predicted protein translation as shown in Figure 2. The remaining two SNPs were translationally-synonymous. Out of 194 amino-acids (AA) translation, five AA differed from the closest species: two of non-conservative groups; two are of semi-conservative groups; and one is conservative. The BLAST analysis and multiple sequence alignment analysis of the *gltA* gene sequences of these three samples demonstrated the same identity seen between these three samples, as shown for the *rOmpA* gene, despite containing less species-specific SNPs compared to other species (Figure S3). Overall, these comparisons have revealed a new sequence segments for both *rOmpA* and *gltA* genes, which had no 100% identity to any other SFG species (Figure 2).

## 3. Discussion

The *R. conorii* group, the etiologic agent of Mediterranean spotted fever, includes several distinct subspecies: the *Malish*, *Indian tick typhus rickettsia* (ITTR), *Israeli spotted fever rickettsia* (ISFR), and *Astrakhan fever rickettsia* (AFR). These strains are closely related by genotype (four MLST genotypes with pairwise similarity in nucleotide sequence that varies from 98.2% to 100%), but differ serotypically and cause diseases with distinct clinical features [15].

For example, *R. conorii israelensis*, which is endemic to Israel [16], has a higher ICU-admission and case-fatality rates than the *R. conorii Malish* strain (29% vs. 13%; 36% vs. 22%) [17]. In addition, the infections of the different strains often manifest clinically different according to strain (for example, *R. israelensis* usually manifests as a fever disease with maculopapular rashes and may result in multi-organ failure and acute encephalitis [18]; *R. slovaca* is implicated in development of tick-borne lymphadenopathy (TIBOLA) or dermacentor-borne necrosis erythema and lymphadenopathy (DEBONEL) [19]; *Rickettsia sibirica* causes lymphadenopathy and lymphangitis [20]; while *Rickesttsia caspia* infections are characterized by febrile illness and a generalized maculopapular rash [15]). These clinical distinctions according to strain constitute the motivation and significance of our finding.

In the current study, we have discovered a new rickettsia isolate from clinical samples of three independent patients with SFG rickettsiosis, based on sequence analysis of three genes that are highly conserved within the group. We identified novel SNP mutations in the *rOmpA* and *gltA* genes, common to these samples. The sequenced fragments of this isolate showed no identity to any other known species documented in the NCBI database. Within a sequence of 194 predicted amino acids, five have no similarity to any of the species that show the highest identity (*caspia*, *conorii israelensis*, *slovaca*, *sibirica*). These SNP mutations were identified in the *rOmpA* and *gltA* genes.

Rickettsial pathogens produce two conserved immunodominant outer membrane proteins, rOmpA and rOmpB, which are thought to be essential for pathogenesis. Mutations in these genes may affect virulence, as previously demonstrated by comparing virulent *R. rickettsii* strains; in which rOmpA is present with the avirulent strain *Iowa*, in which a premature stop codon prevents its production [21]. Together, these clinical samples (from patients admitted to three different Hospitals in Israel, from distinct geographic locations, and temporally separated by one to two years from each other) establish the basis for the identification of a previously unknown new isolate that has clinical significance. Further research should focus on the endemic prevalence and distribution of this new isolate in Israel. This may be carried out by epidemiologic mapping of Israeli tick reservoir and routine molecular studying of all clinical cases of SFG in Israel.

This new isolate may also cause a distinct type of clinical presentation and outcome, possibly explaining the varying outcomes observed in patients with SFG rickettsiosis, not always explained strictly by host factors. Therefore, this new SFG rickettsia should be familiar to both physicians and researchers, since there are clinical and epidemiological implications on initial diagnosis, treatment and outcomes.

## 4. Materials and Methods 

PCR amplification

Genomic DNA was extracted from clinical samples using the QIAamp DNA Mini Kit (Qiagen cat.51304) according to manufacturer’s instructions.

Diagnostic nested PCR and differentiating digestion were carried out as previously described [22].

Further PCR reactions were performed separately for each sample, using the oligonucleotide primers listed in Table 2. Most of the primers were designed according to conserved regions after alignment of about 15 SFG rickettsia species.

Five microliters (µL) of the extracted DNA were added to a 50 µL reaction mixture, containing: 5 µL of 5 picomol/µL of each primer, 10 µL of MyTaqTM 5x Buffer (Bioline cat.BIO-21111), 0.4 µL enzyme MyTaqTM (Bioline cat.BIO-21111) and 24.6 µL DDW.

The following conditions were used for amplification: initial denaturation for 3 min at 94 °C, followed by 40 cycles of annealing for 30 sec at 55 °C, extension for 1 min and 30 sec at 72 °C, denaturation for 30 sec at 94 °C. After these cycles, a final cycle of annealing for 30 sec at 55 °C, then 5 min at 72 °C to allow complete extension of the PCR products was performed.

PCR reactions were carried out in a T100TM thermal cycler (BIO-RAD, 1861096).

Sequencing reaction:

Sequencing using the same primers as used for amplification, was carried out by Hy Laboratories Ltd (hylabs) using an ABI 3730xl DNA Analyzer. Each sample was sequenced separately.

Data analysis:

The sequences used in this study were approved following a process of meticulous manual examination, looking into the quality of the specific peaks of the SNPs described. In addition, the fragments were separately sequenced on both directions, yielding 100% homologous results. Data was analyzed using the BioEdit Sequence Alignment Editor. BLAST was performed using NCBI BLAST (National Center for Biotechnology Information)–BLASTN for nucleotide BLAST or BLASTX for protein BLAST. Sequences were aligned using EMBL-EBI T-Coffee MSA tool.

Ethical statement:

All subjects gave their informed consent for inclusion before they participated in the study. The study was conducted in accordance with the Declaration of Helsinki, and the protocol was approved by the Ethics Committee of The Sanz Medical Center, Lanaido Hospital, Netanya (Project identification codes 0002-19-LND).

## Figures and Tables

**Table 1:** Percent identity matrix for *rOmpA* sequenced segment—created by Clustal2.1 *.

	Patient 1	Patient 2	Patient 3	*Rickettsia caspia*	*Rickettsia conorii israelensis*	*Rickettsia sibirica*	*Rickettsia slovaca*
**Patient 1**	-	100	100	96.98	95.34	92.23	95.85
**Patient 2**	100	-	100	96.89	95.34	92.23	95.85
**Patient 3**	100	100	-	96.89	95.34	92.23	95.85
***Rickettsia caspia***	96.91	96.91	96.91	-	97.44	93.81	97.94
***Rickettsia conorii israelensis***	95.36	95.36	95.36	97.44	-	92.27	95.36
***Rickettsia sibirica***	92.27	92.27	92.27	93.81	92.27	-	95.36
***Rickettsia slovaca***	95.88	95.88	95.88	97.94	95.36	95.36	-

* The species to which each patient sequence was compared to, were *R. caspia* (GenBank accession number U83437.1), *R. conorii Israelensis* (GenBank accession number U83441.1), *R. sibirica* (GenBank accession number U83455.1) and *R. slovaca* (GenBank accession number JX683121.1).

**Table 2:** Oligonucleotide primers used for PCR amplification and sequencing of *Rickettsia* species.

Primer Name	Target Gene	Primer Sequence 5′-3′
213F	*OmpA*	AATCAATATTGGAGCCGGTAA
667R	*OmpA*	ATTTGCATCAATCGTATAAGTAGC
120F	*OmpA*	AAGGAGCTATAGCAAACGGCA
760R	*OmpA*	TATCAGGGTCTATATTCGCACCTA
760newF	*OmpA*	TAGGTGCGAATATAGACCCTGATA
1231R	*OmpA*	TGGCAATAGTTACATTTCCTGCAC
373F	*gltA*	TTGTAGCTCTTCTCATCCTATGGC
1138R	*gltA*	CATTTGCGACGGTATACCCATA
Rico173F	*gltA*	CGACCCGGGTTTTATGTCTA ^1^
1179R	*gltA*	TCCAGCCTACGATTCTTGCTA

^1^ All primers were design in our lab, except for Primer Rico173F [2].

## Supplementary Materials

The following are available online at https://www.mdpi.com/2076-0817/9/1/11/s1, Figure S1: Phylogenetic tree analysis of the new isolate along known rickettsial species according to: A. *rOmpA*, B. *gltA*, Figure S2: Multiple sequence alignment, for the *rOmpA* nucleotide sequence of the three clinical samples (patients 1–3) and four known rickettsia species, Figure S3: Multiple sequence alignment, for the *gltA* nucleotide sequence of the three clinical samples (patients 1–3) and four known rickettsia species.
