# Supplementary material for: New Spotted Fever Group Rickettsia Isolate, Identified by Sequence Analysis of Conserved Genomic Regions
Source: Pathogens. 2019 Dec 20;9(1):11. doi: 10.3390/pathogens9010011 (PMC7168670; doi:10.3390/pathogens9010011)
Supplement: Supplementary file 1 [file pathogens-09-00011-s001.pdf]

pathogens

MDPI

A.

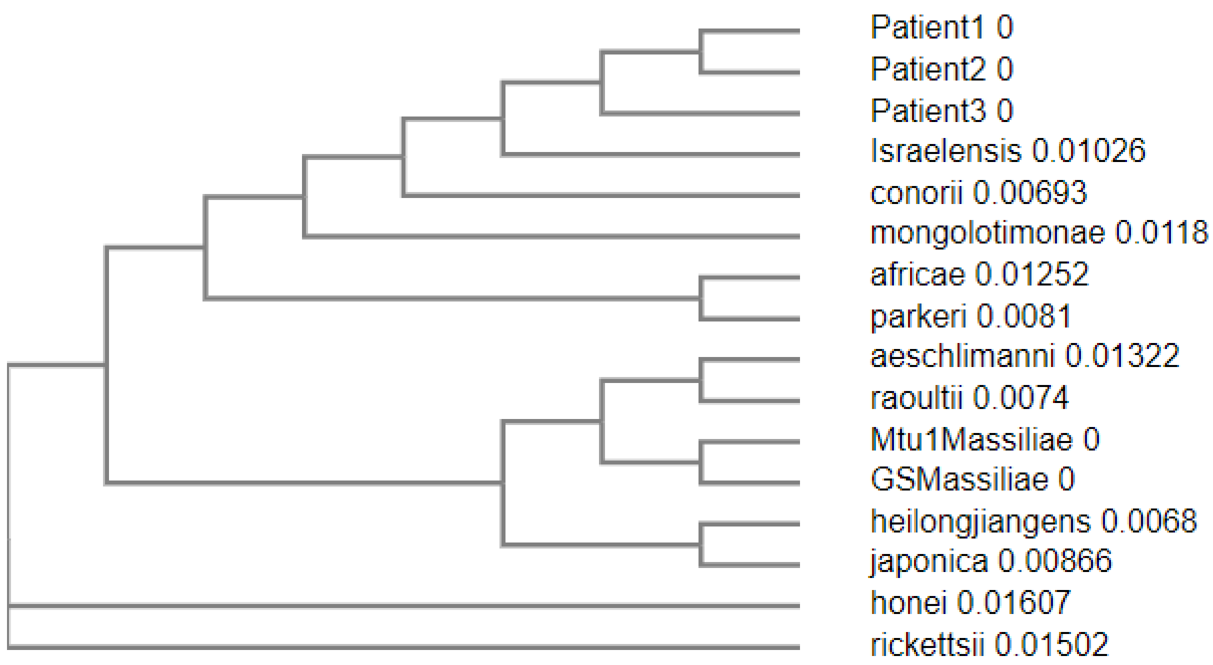

B.

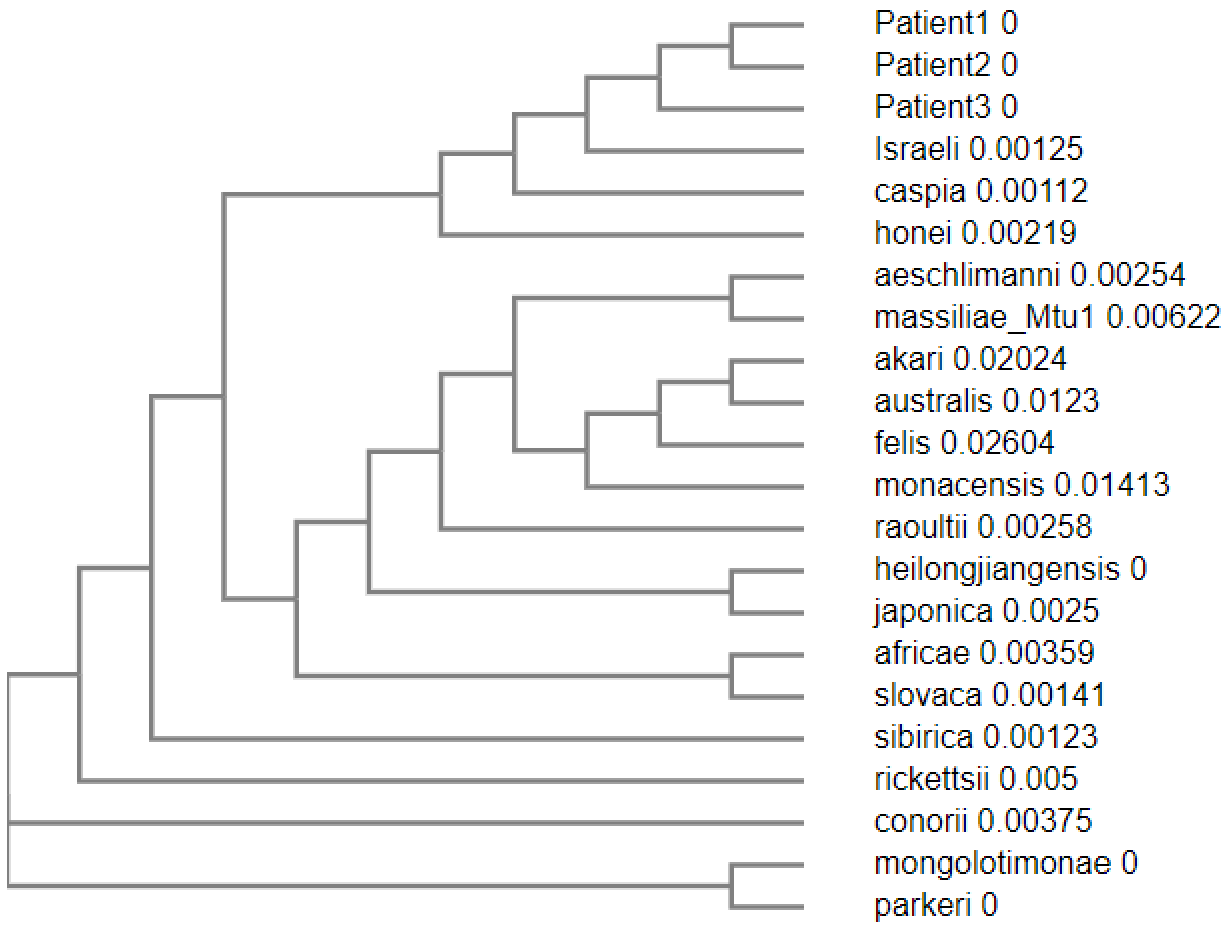

Figure S1. Phylogenetic tree analysis of the new isolate along known rickettsial species according to: A. rOmpA, B. gltA.

Phylogenetic analysis was performed using the EMBL-EBI Simple Phylogeny tool:

```
Patient #1    TATATTAAATGTTAATACAAAGTTACTTACGGCATCTCATTTAACTATAGGAACAGTTGCAGAAATCAA
Patient #2    TATATTAAATGTTAATACAAAGTTACTTACGGCATCTCATTTAACTATAGGAACAGTTGCAGAAATCAA
Patient #3    TATATTAAATGTTAATACAAAGTTACTTACGGCATCTCATTTAACTATAGGAACAGTTGCAGAAATCAA
Caspia        TATATTAAATGTTAATACAAAGTTACTTACGGCATATCATTTAACTATAGGAACAGTTGCAAAAATCAA
Israelensis   TATATTAAATGTTAATACAAAGTTACTTACGGCATATCATTTAACTATAGGAACAGTTGCAAAAATCAA
Sibirica      TATATTAAATGTTAATACAAAGTTACTTACGGCATATCATTTAACTATAGGAACAGTTGCAGAAATCAA
Slovaca       TATATTAAATGTTAATACAAAGTTACTTACGGCATATCATTTAACTATAGGAACAGTTGCAGAAATCAA
cons          *********************************** ************************* *******

Patient #1    TATTGGAGCCGGTAATTTTTTTGCAATTGATGCAAGTGCTGGTGATGTTACTATATTAAAT---GCTCA
Patient #2    TATTGGAGCCGGTAATTTTTTTGCAATTGATGCAAGTGCTGGTGATGTTACTATATTAAAT---GCTCA
Patient #3    TATTGGAGCCGGTAATTTTTTTGCAATTGATGCAAGTGCTGGTGATGTTACTATATTAAAT---GCTCA
Caspia        TATTGGAGCCGGTAATTTTTTTGCAATTGATGCAAGTGCTGGTGATGTTACTATATTAAATAATGCTCA
Israelensis   TATTGGAGCCGGTAATTTTTTTGCAATTGATGCAAGTGCTGGTGATGTTACTATATTAAATAATGCTCA
Sibirica      TATTGGAGCCGGTAATATTTTTGCAATTGATGCAAGTGCTGGTGATGTTACTATATTAAAT---GCTCA
Slovaca       TATTGGAGCCGGTAATCTTTTTGCAATTGATGCAAGTGCTGGTGATGTTACTATATTAAAT---GCTCA
cons          **************** ********************************************   *****

Patient #1    AGATATTCATTTAGAGCTCTAGATTCTGCTTTAGTACTTTCTAACTTAGCCGGAGTCGGAGTAAATAA
Patient #2    AGATATTCATTTAGAGCTCTAGATTCTGCTTTAGTACTTTCTAACTTAGCCGGAGTCGGAGTAAATAA
Patient #3    AGATATTCATTTAGAGCTCTAGATTCTGCTTTAGTACTTTCTAACTTAGCCGGAGTCGGAGTAAATAA
Caspia        AGATATTCATTTAGAGCTCTAGATTCTGCTTTAGTACTTTCTAACTTAACCGGAGTCGGAGTAAATAA
Israelensis   AGATATTCATTTAGAGCTCTAGATTCTGCTTTAGTACTTTCTAACTTAACCGGAGCCGGAGTAAATAA
Sibirica      AGATATTCATTTAGAGCTCTAGATTCTACTTTAGTACTTTCTAACTTAACCGGAGTCGGAGTAAATAA
Slovaca       AGATATTCATTTAGAGCTCTAGATTCTGCTTTAGTACTTTCTAACTTAACCGGAGTCGGAGTAAATAA
cons          *************************** ******************** ****** ************

Patient #1    TATATTATTAGCAGCTGATTTAGTAGCTCCCGGTGCTGCTGAAGGTACGGTAGTCTTTGATGGTGGGGT
Patient #2    TATATTATTAGCAGCTGATTTAGTAGCTCCCGGTGCTGCTGAAGGTACGGTAGTCTTTGATGGTGGGGT
Patient #3    TATATTATTAGCAGCTGATTTAGTAGCTCCCGGTGCTGCTGAAGGTACGGTAGTCTTTGATGGTGGGGT
Caspia        TATATTATTAGCAGCTGATTTAGTAGCTCCCGGTGCTGATGAAGGTACGGTAGTCTTTGATGGTGGGGT
Israelensis   TATATTATTAGCAGCTGATTTAGTAGCTCCCGGTGCTGATGAAGGTACGGTAGTCTTTGATGGTGGGGT
Sibirica      TATATTATTAGCAGCTGATTTAGTAGCTCCCGGTGCTGGTGAAGGTACGGTAATCTTTAATGGTGGGGT
Slovaca       TATATTATTAGCAGCTGATTTAGTAGCTCCCGGTGCTGATGAAGGTACGGTAGTCTTTAATGGTGGGGT
cons          ************************************** ************* ***** **********

Patient #1    TAATGGTCTGAATATTGGGAGTAATGTAGCAGGTACCGCTAGAAATATCGGTGATGGAGGCGGTAATAA
Patient #2    TAATGGTCTGAATATTGGGAGTAATGTAGCAGGTACCGCTAGAAATATCGGTGATGGAGGCGGTAATAA
Patient #3    TAATGGTCTGAATATTGGGAGTAATGTAGCAGGTACCGCTAGAAATATCGGTGATGGAGGCGGTAATAA
Caspia        TAATGGTCTGAATATTGGGAGTAATGTAGCAGGTACCGCTAGAAATATCGGTGATGGAGGCGGTAATAA
Israelensis   TAATGGTCTGAATATTGGGAGTAATGTAGCAGGTACCGCTAGAAATATCGGTGATGGAGGCGGTAATAA
Sibirica      TAATGGTCTGCATATTGGGAGTAATGTAGCAGGTACCGCTAGAAATATCGGTGATGGAGGCGGTAATAA
Slovaca       TAATGGCCTGAATATTGGGAGTAATGTAGCAGGTACCGCTAGAAATATCGGTGATGGAGGCGGTAATAA
cons          ****** *** **********************************************************

Patient #1    ATTTAACACTTTACTTATTTATAATGCCGTTACAATAACTGACGATGTAAATTTAGAAGGTATACAGAA
Patient #2    ATTTAACACTTTACTTATTTATAATGCCGTTACAATAACTGACGATGTAAATTTAGAAGGTATACAGAA
Patient #3    ATTTAACACTTTACTTATTTATAATGCCGTTACAATAACTGACGATGTAAATTTAGAAGGTATACAGAA
Caspia        ATTTAACACTTTACTTATTTATAATGCCGTTACAATAACTGACGATGTAAATTTAGAAGGTATACAGAA
Israelensis   ATTTAACACTTTACTTATTTATAATGCCGTTACAATAACTGACGATGTAAATTTAGAAGGTATACAGAA
Sibirica      ATTTAACGCTTTACTTATTTATAATGCCGTTACAATAACTGACGATGTAAATTTAGAAGGTATACAGAA
Slovaca       ATTTAACACTTTACTTATTTATAATGCCGTTACAATAACTGACGATGTAAATTTAGAAGGTATACAGAA
cons          ******* *************************************************************

Patient #1    CGTGCTTATTAACAATAATGCAGGTTTTACTAGTAGTACAGCATTTAATGCTGGTGCTATACAAATAAA
```

```
Patient #2     CGTGCTTATTAACAATAATGCAGGTTTTACTAGTAGTACAGCATTTAATGCTGGTGCTATACAAATAAA
Patient #3     CGTGCTTATTAACAATAATGCAGGTTTTACTAGTAGTACAGCATTTAATGCTGGTGCTATACAAATAAA
Caspia         CGTGCTTATTAACAATAATGCAGATTTTACTAGTAGTACAGCATTTAATGCTGGTGCTATACAAATAAA
Israelensis    CGTGCTTATTAACAATAATGCAAATTTTACTAGTAGTACAGCATTTGATGCTGGTGCTATACAAATAAA
Sibirica       CGTGCTTATTAACAATAATGCAGATTTTACTAGTAGTACAGCATTTAATGCTGGTGTTATACAAATACA
Slovaca        CGTGCTTATTAACAATAATGCAGATTTTACTAGTAGTACAGCATTTAATGCTGGTGCTATACAAATAAA
cons           **********************  ********************** ********* ********** *

Patient #1     CGATGCTACTTATACGATTGATGCAAATAATGGTAATTTAAATATACCGGCAGGAAATATTAAATTTGC
Patient #2     CGATGCTACTTATACGATTGATGCAAATAATGGTAATTTAAATATACCGGCAGGAAATATTAAATTTGC
Patient #3     CGATGCTACTTATACGATTGATGCAAATAATGGTAATTTAAATATACCGGCAGGAAATATTAAATTTGC
Caspia         CGATGCTACTTATACGATTGATGCAAATAATGGTAATTTAAATATACCGGCAGGAAATATTAAATTTGC
Israelensis    CAATGCTACTTATACGATTGATGCAAATAATGGTAATTTAAATATACCGGCAGGAAATATTAAATTTGC
Sibirica       AAATGCTACTTATACGATTGATGCAAATAATGGTAATTTAAATATACCGGCAGGAAATATTCAATTTGC
Slovaca        CGATGCTACTTATACGATTGATGCAAATAATGGTAATTTAAATATACCGGCAGGAAATATTCAATTTGC
cons             *********************************************************** *******

Patient #1     ACATGCGGGTGCTCAATTAATATTACAAAATAGT
Patient #2     ACATGCGGGTGCTCAATTAATATTACAAAATAGT
Patient #3     ACATGCGGGTGCTCAATTAATATTACAAAATAGT
Caspia         ACATGCGGATGCTCAATTAATATTACAAAATAGT
Israelensis    ACATGCGAATGCTCAATTAATATTACAAAATAGT
Sibirica       ACATGCGGATGCTCAATTAATATTACAAAATAGT
Slovaca        ACATGCGGATGCTCAATTAATATTACAAAATAGT
cons           *******  *************************
```

Figure S2. Multiple sequence alignment, for the rOmpA nucleotide sequence of the three clinical samples (patients 1-3) and four known rickettsia species. The alignment was performed using EMBL-EBI Clustal W (1.83) with forward primer 120F and Reversed primer 760R. R. caspia (GenBank accession number U83437.1), R. conorii Israelensis (GenBank accession number U83441.1), R. sibirica (GenBank accession number U83455.1) and R. slovaca (GenBank accession number JX683121.1). Yellow Highlighted = mutations unique to the clinical samples, not appear in any known rickettsia species. Orange Highlighted = identical nucleotides- showing similarity or difference between the clinical samples and the rickettsia species.

```
Patient #1    CAAGTGGCGAGCAGTATAATAATTTCACTAAACAGGTTGCTCATCATTCATTAGTGAATGAAAGATTAC
Patient #2    CAAGTGGCGAGCAGTATAATAATTTCACTAAACAGGTTGCTCATCATTCATTAGTGAATGAAAGATTAC
Patient #3    CAAGTGGCGAGCAGTATAATAATTTCACTAAACAGGTTGCTCATCATTCATTAGTGAATGAAAGATTAC
caspia        CAAGTGGCGAGCAGTATAATAATTTCACTAAACAGGTTGCTCATCATTCATTAGTGAATGAAAGATTAC
Israelensis   CAAGTGGCGAGCAGTATAATAATTTCACTAAACAGGTTGCTCATCATTCATTAGTGAATGAAAGATTAC
sibirica      CAAGTGGCGAGCAGTATAATAATTTCACTAAACAGGTTGCTCATCATTCATTAGTGAATGAAAGATTAC
slovaca       CAAGTGGTGAGCAGTATAATAATTTCACTAAACAGGTTGCTCATCATTCATTAGTGAATGAAAGATTAC
cons          ******* *************************************************************

Patient #1    ACTATTTATTTCAAACCTTTTGTAGCTCTTCTCATCCTATGGCTATTATGCTTGCGGCTGTCGGTTCTC
Patient #2    ACTATTTATTTCAAACCTTTTGTAGCTCTTCTCATCCTATGGCTATTATGCTTGCGGCTGTCGGTTCTC
Patient #3    ACTATTTATTTCAAACCTTTTGTAGCTCTTCTCATCCTATGGCTATTATGCTTGCGGCTGTCGGTTCTC
caspia        ACTATTTATTTCAAACCTTTTGTAGCTCTTCTCATCCTATGGCTATTATGCTTGCGGCTGTCGGTTCTC
Israelensis   ACTATTTATTTCAAACCTTTTGTAGCTCTTCTCATCCTATGGCTATTATGCTTGCGGCTGTCGGTTCTC
sibirica      ACTATTTATTTCAAACCTTTTGTAGCTCTTCTCATCCTATGGCTATTATGCTTGCGGCTGTCGGTTCTC
slovaca       ACTATTTATTTCAAACCTTTTGTAGCTCTTCTCATCCTATGGCTATTATGCTTGCGGCTGTCGGTTCTC
cons          *********************************************************************

Patient #1    TTTCGGCATTTTATCCTGATTTATTGAATTTTAAGGAAGCAGATTACGAACTTACCGCTATTAGAATGA
Patient #2    TTTCGGCATTTTATCCTGATTTATTGAATTTTAAGGAAGCAGATTACGAACTTACCGCTATTAGAATGA
Patient #3    TTTCGGCATTTTATCCTGATTTATTGAATTTTAAGGAAGCAGATTACGAACTTACCGCTATTAGAATGA
caspia        TTTCGGCATTTTATCCTGATTTATTGAATTTTAAGGAAGCAGATTACGAACTTACCGCTATTAGAATGA
Israelensis   TTTCGGCATTTTATCCTGATTTATTGAATTTTAAGGAAGCAGATTACGAACTTACCGCTATTAGAATGA
sibirica      TTTCGGCATTTTATCCTGATTTATTGAATTTTAAGGAAGCAGATTACGAACTTACCGCTATTAGAATGA
slovaca       TTTCGGCATTTTATCCTGATTTATTGAATTTTAAGGAAGCAGATTACGAACTTACCGCTATTAGAATGA
cons          *********************************************************************

Patient #1    TTGCTAAGATACCTACCATCGCTGCAATGTCTTATAAATATTCTATAGGACAACCGTTTATTTATCCTG
Patient #2    TTGCTAAGATACCTACCATCGCTGCAATGTCTTATAAATATTCTATAGGACAACCGTTTATTTATCCTG
Patient #3    TTGCTAAGATACCTACCATCGCTGCAATGTCTTATAAATATTCTATAGGACAACCGTTTATTTATCCTG
caspia        TTGCTAAGATACCTACCATCGCTGCAATGTCTTATAAATATTCTATAGGACAACCGTTTATTTATCCTG
Israelensis   TTGCTAAGATACCTACCATCGCTGCAATGTCTTATAAATATTCTATAGGACAACCGTTTATTTATCCTG
sibirica      TTGCTAAGATACCTACCATCGCTGCAATGTCTTATAAATATTCTATAGGACAACCGTTTATTTATCCTG
slovaca       TTGCTAAGATACCTACCATCGCTGCAATGTCTTATAAATATTCTATAGGACAACCGTTTATTTATCCTG
cons          *********************************************************************

Patient #1    ATAATTCGTTAGATTTTACCGAAAATTTTCTGCATATGATGTTTGCAACGCCTTGTACGAAATATACAG
Patient #2    ATAATTCGTTAGATTTTACCGAAAATTTTCTGCATATGATGTTTGCAACGCCTTGTACGAAATATACAG
Patient #3    ATAATTCGTTAGATTTTACCGAAAATTTTCTGCATATGATGTTTGCAACGCCTTGTACGAAATATACAG
caspia        ATAATCCGTTAGATTTTACCGAAAATTTTCTGCATATGATGTTTGCAACGCCTTGTACGAAATATACAG
Israelensis   ATAATTCGTTAGATTTTACCGAAAATTTTCTGCATATGATGTTTGCAACGCCTTGTACGAAATATACAG
sibirica      ATAATTCGTTAGATTTTACCGAAAATTTTCTGCATATGATGTTTGCAACGCCTTGTACGAAATATACAG
slovaca       ATAATTCGTTAGATTTTACCGAAAATTTTCTGCATATGATGTTTGCAACGCCTTGTACGAAATATACAG
cons          ***** ***************************************************************

Patient #1    TAAATCCAATAATAAAAAATGCTCTTAATAAGATATTTATCCTACATGCCGATCATGAGCAGAATGCTT
Patient #2    TAAATCCAATAATAAAAAATGCTCTTAATAAGATATTTATCCTACATGCCGATCATGAGCAGAATGCTT
Patient #3    TAAATCCAATAATAAAAAATGCTCTTAATAAGATATTTATCCTACATGCCGATCATGAGCAGAATGCTT
caspia        TAAATCCAATAATAAAAAATGCTCTTAATAAGATATTTATCCTACATGCCGATCATGAGCAGAATGCTT
Israelensis   TAAATCCAATAATAAAAAATGCTCTTAATAAGATATTTATCCTACATGCCGATCATGAGCAGAATGCTT
sibirica      TAAATCCAATAATAAAAAATGCTCTTAATAAGATATTTATCCTACATGCCGATCATGAGCAGAATGCTT
slovaca       TAAATCCAATAATAAAAAATGCTCTTAATAAGATATTTATCCTACATGCCGATCATGAGCAGAATGCTT
cons          *********************************************************************

Patient #1    CTACTTCAACAGTCCGAATTGCCGGCTCATCCGGAGCTAACCCTTTTGCTTGTATTAGCACGGGTATTG
```

```
Patient #2    CTACTTCAACAGTCCGAATTGCCGGCTCATCCGGAGCTAACCCTTTTGCTTGTATTAGCACGGGTATTG
Patient #3    CTACTTCAACAGTCCGAATTGCCGGCTCATCCGGAGCTAACCCTTTTGCTTGTATTAGCACGGGTATTG
caspia        CTACTTCAACAGTCCGAATTGCCGGCTCATCCGGAGCTAACCCTTTTGCTTGTATTAGCACGGGTATTG
Israelensis   CTACTTCAACAGTCCGAATTGCCGGCTCATCCGGAGCTAACCCTTTTGCTTGTATTAGCACGGGTATTG
sibirica      CTACTTCAACAGTCCGAATTGCCGGCTCATCCGGAGCTAACCCTTTTGCTTGTATTAGCACGGGTATTG
slovaca       CTACTTCAACAGTCCGAATTGCCGGCTCATCCGGAGCTAACCCTTTTGCTTGTATTAGCACGGGTATTG
cons          *********************************************************************

Patient #1    CCTCACTTTGGGGACCTGCTCACGGCGGGGCTAATGAAGCGGTAATAAATATGCTTAAAGAAATCGGTA
Patient #2    CCTCACTTTGGGGACCTGCTCACGGCGGGGCTAATGAAGCGGTAATAAATATGCTTAAAGAAATCGGTA
Patient #3    CCTCACTTTGGGGACCTGCTCACGGCGGGGCTAATGAAGCGGTAATAAATATGCTTAAAGAAATCGGTA
caspia        CCTCACTTTGGGGACCTGCTCACGGCGGGGCTAATGAAGCGGTAATAAATATGCTTAAAGAAATCGGTA
Israelensis   CCTCACTTTGGGGACCTGCTCACGGCGGGGCTAATGAAGCGGTAATAAATATGCTTAAAGAAATCGGTA
sibirica      CCTCACTTTGGGGACCTGCTCACGGCGGGGCTAATGAAGCGGTAATAAATATGCTTAAAGAAATCGGTA
slovaca       CCTCACTTTGGGGACCTGCTCACGGCGGGGCTAATGAAGCGGTAATAAATATGCTTAAAGAAATCGGTA
cons          *********************************************************************

Patient #1    GTTCTGAGTATATTCCTAAATATATAGCTAAAGCTAAGGATAAAAATGATCCATTTAGATTAATGGGTT
Patient #2    GTTCTGAGTATATTCCTAAATATATAGCTAAAGCTAAGGATAAAAATGATCCATTTAGATTAATGGGTT
Patient #3    GTTCTGAGTATATTCCTAAATATATAGCTAAAGCTAAGGATAAAAATGATCCATTTAGATTAATGGGTT
caspia        GTTCTGAGTATATTCCTAAATATATAGCTAAAGCTAAGGATAAAAATGATCCATTTAGATTAATGGGTT
Israelensis   GTTCTGAGTATATTCCTAAATATATAGCTAAAGCTAAGGATAAAAATGATCCATTTAGATTAATGGGTT
sibirica      GTTCTGAGTATATTCCTAAATATATAGCTAAAGCTAAGGATAAAAATGATCCATTTAGATTAATGGGTT
slovaca       GTTCTGAGTATATTCCTAAATATATAGCTAAAGCTAAGGATAAAAATGATCCATTTAGATTAATGGGTT
cons          *********************************************************************

Patient #1    TTGGTCATCGTGTATATAAAAACTATGACCCGCGTGCCGCAGTACTTAAAGAAACGTGCAAAGAAGTAT
Patient #2    TTGGTCATCGTGTATATAAAAACTATGACCCGCGTGCCGCAGTACTTAAAGAAACGTGCAAAGAAGTAT
Patient #3    TTGGTCATCGTGTATATAAAAACTATGACCCGCGTGCCGCAGTACTTAAAGAAACGTGCAAAGAAGTAT
caspia        TTGGTCATCGTGTATATAAAAACTATGACCCGCGTGCCGCAGTACTTAAAGAAACGTGCAAAGAAGTAT
Israelensis   TTGGTCATCGTGTATATAAAAACTATGACCCGCGTGCCGCAGTACTTAAAGAAACGTGCAAAGAAGTAT
sibirica      TTGGTCATCGTGTATATAAAAACTATGACCCGCGTGCCGCAGTACTTAAAGAAACGTGCAAAGAAGTAT
slovaca       TTGGTCATCGTGTATATAAAAACTATGACCCGCGTGCCGCAGTACTTAAAGAAACGTGCAAAGAAGTAT
cons          *********************************************************************

Patient #1    TAAAGGCACTCGGGCAGCTAGACAACAATCCGCTCTTACAAATAGCAATAGAACTTGAAGCTATCGCTC
Patient #2    TAAAGGCACTCGGGCAGCTAGACAACAATCCGCTCTTACAAATAGCAATAGAACTTGAAGCTATCGCTC
Patient #3    TAAAGGCACTCGGGCAGCTAGACAACAATCCGCTCTTACAAATAGCAATAGAACTTGAAGCTATCGCTC
caspia        TAAAGGCACTCGGGCAGCTAGACAACAATCCGCTCTTACAAATAGCAATAGAACTTGAAGCTATCGCTC
Israelensis   TAAAGGCACTTGGGCAGCTAGACAACAATCCGCTCTTACAAATAGCAATAGAACTTGAAGCTATCGCTC
sibirica      TAAAGGAACTCGGGCAGCTAGACAACAATCCGCTCTTACAAATAGCAATAGAACTTGAAGCTATCGCTC
slovaca       TAAAGGAACTCGGGCAGCTAGACAACAATCCGCTCTTACAAATAGCAATAGAACTTGAAGCTATTGCTC
cons          ****** *** ***************************************************** ****

Patient #1    TTAAAGATGAATATTTTATTGAGAGAAAATTATATCC
Patient #2    TTAAAGATGAATATTTTATTGAGAGAAAATTATATCC
Patient #3    TTAAAGATGAATATTTTATTGAGAGAAAATTATATCC
caspia        TTAAAGATGAATATTTTATTGAGAGAAAATTATATCC
Israelensis   TTAAAGATGAATATTTTATTGAGAGAAAATTATATCC
sibirica      TTAAAGATGAATATTTTATTGAGAGAAAATTATATCC
slovaca       TTAAAGATGAATATTTTATTGAGAGAAAATTATATCC
cons          *************************************
```

Figure S3. Multiple sequence alignment, for the gltA nucleotide sequence of the three clinical samples (patients 1-3) and four known rickettsia species. The alignment was performed using EMBL-EBI Clustal W (1.83) with forward primer

rico173F and Reversed primer 1179R. *R. caspia* (GenBank accession number U59728.1), *R. conorii Israelensis* (GenBank accession number U59727.1), *R. sibirica* (GenBank accession number KM288711.1) and *R. slovaca* (GenBank accession number DQ176434.1). Yellow Highlighted = mutations unique to the clinical samples, not appear in any known rickettsia species. Orange Highlighted = identical nucleotides- showing similarity or difference between the clinical samples and the rickettsia species.
